# Effect of Filtration and Slice Thickness of Cone-Beam Computed Tomography Images on Occlusal Caries Detection: An Ex Vivo Study

**Published:** 2018-09

**Authors:** Mehrdad Abdinian, Marzieh Ghaiour

**Affiliations:** 1 Associate Professor, Dental Implant Research Center, Department of Oral and Maxillofacial Radiology, School of Dentistry, Isfahan University of Medical Sciences, Isfahan, Iran; 2 Postgraduate Student, Department of Pediatric Dentistry, School of Dentistry, Isfahan University of Medical Sciences, Isfahan, Iran

**Keywords:** Biomedical Research, Cone-Beam Computed Tomography, Dental Caries, Filtration

## Abstract

**Objectives:**

The aim of this study was to evaluate the diagnostic accuracy of different filtrations and slice thicknesses of cone-beam computed tomography (CBCT) in the detection of occlusal caries.

**Materials and Methods:**

One-hundred teeth were selected for this ex-vivo experimental study. The CBCT images of the teeth were evaluated and scored by two observers in panoramic and cross-sectional views using different slice thicknesses and filtrations. Paired t-test, repeated-measures analysis of variance (ANOVA), and the least significant difference (LSD) test were used to compare the data with the histological gold standard. Receiver operating characteristic (ROC) analysis was used to determine the diagnostic accuracy of each slice thickness and filtration (P<0.05).

**Results:**

The mean score of true caries detection in cross-sectional views was lower than that in panoramic views (P<0.05). Repeated-measures ANOVA showed a significant difference in the mean of true detections in different thicknesses of cross-sectional views, but this difference was significant only between 5 mm thickness and other thicknesses in panoramic views. On all the views, increasing the thickness decreased the accuracy of caries detection. Repeated-measures ANOVA showed a significant difference between different filtrations; on all the views, increasing the filtration increased the accuracy of caries detection.

**Conclusions:**

An increase of filtration of CBCT images increases the accuracy of occlusal caries detection; however, an increase in slice thickness results in a lower diagnostic accuracy.

## INTRODUCTION

Dental caries is the most common problem in dentistry with a high incidence rate in the population [1,2]. The diagnosis of caries requires knowledge about caries depth to reach an appropriate treatment plan [3,4].

Clinical examination and intraoral radiography are the most common methods for the detection of dental caries. Several studies have demonstrated that 25% to 42% of carious cases are not detected with clinical examination alone [5,6], and no single method will allow the detection of caries on all tooth surfaces [7]; therefore, radiography is an important tool for caries detection. Radiographic methods for caries detection were limited to conventional radiography, charge-coupled devices (CCD), and photostimulable phosphor plates (PSP) in the past years [8,9].

Cone-beam computed tomography (CBCT) is a new radiographic technique, which is now used in the diagnosis of different diseases and for treatment planning. This technique produces images in the axial, sagittal, and coronal planes, and has the ability to produce three-dimensional (3D) images. Some advantages of CBCT are high accuracy, lower artifact, lower exposure dose, and lower scanning time compared to CT [10–12].

Recent studies have evaluated the caries detection accuracy of CBCT; however, the effectiveness of CBCT in this respect is controversial [13–16]. The detection of occlusal caries with radiography is more complicated due to the complexity of occlusal surface anatomy [17]. The effect of filtration on the accuracy of digital radiographs is still controversial. One study demonstrated that filtration masks root fractures and decreases the diagnostic accuracy [18].

If a patient has a CBCT scan obtained for another treatment plan, it is possible to evaluate the existence of caries on this scan, eliminating the need for other radiographic techniques, provided that CBCT imaging is proven accurate enough for caries detection. This is in compliance with the "as low as reasonably achievable" (ALARA) principle since CBCT has a low radiation dose [19]. Therefore, the present study was undertaken to evaluate the effect of filtration and slice thickness of CBCT images on occlusal caries detection.

## MATERIALS AND METHODS

This study was conducted under an approval by the Institutional Review Board of Isfahan University of Medical Sciences (No. 393558).

**Fig. 1:**
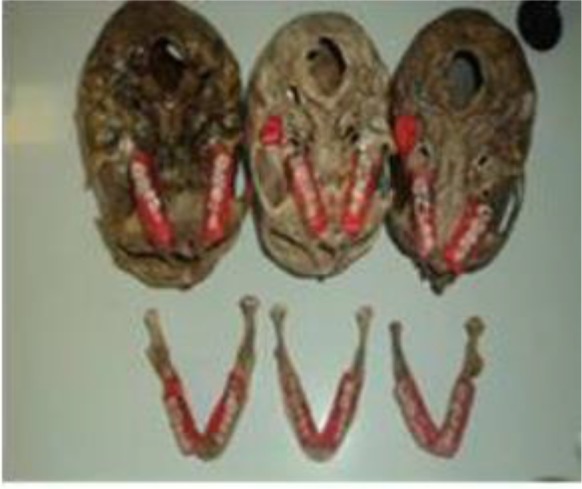
Placement of teeth in a normal anatomical position

In this ex-vivo descriptive study, 100 human teeth including 20 canines, 40 premolars, and 40 molars, with or without occlusal caries, were evaluated. The teeth had been extracted for periodontal or orthodontic reasons at the school of dentistry of Isfahan University of Medical Sciences. The teeth were cleaned of calculi and debris using an ultrasonic device (Cavitron JET Plus Ultrasonic Scaler, Dentsply, Milford, Delaware, USA), disinfected in 2% sodium hypochlorite (NaOCl) solution for 20 minutes, and stored in distilled water. Then, they were sectioned into coronal and radicular pieces using a #835 fissure bur (0.8 mm in diameter; Tizkavan, Tehran, Iran) with an appropriate coolant. The coronal sections of the teeth were itemized into canine, first and second premolars, and first and second molars of the maxilla or mandible. Next, the teeth were randomly divided into 20 groups; 10 groups comprised maxillary teeth (5 teeth on the left side, and 5 teeth on the right side), and 10 groups included mandibular teeth (5 teeth on the left side, and 5 teeth on the right side). Each group contained a canine, first and second premolars, and first and second molars of the same jaw and of the same quadrant. The teeth in each group were placed in appropriate alveolar sockets of a dry human skull with a dry mandible. The placement was carried out such that each row was in proper proximal contacts. The mandibular and maxillary teeth were occluded together and were fixed using wax, simulating a normal anatomical position (Fig. 1).

A 14.5-mm-thick acrylic block was placed in front of the teeth to simulate the soft tissue and its scattering effect [20]. As the next step, the skulls were radiographed using a CBCT system (Soredex, Helsinki, Finland). Each skull was fixed in the CBCT system using ear rods and was exposed to a voltage of 89 kilovoltage peak (kVp) and a tube current of 6 milliamperes (mA) with a scanning time of 12.6 seconds in an 8-cm field of view (FOV).

**Fig. 2:**
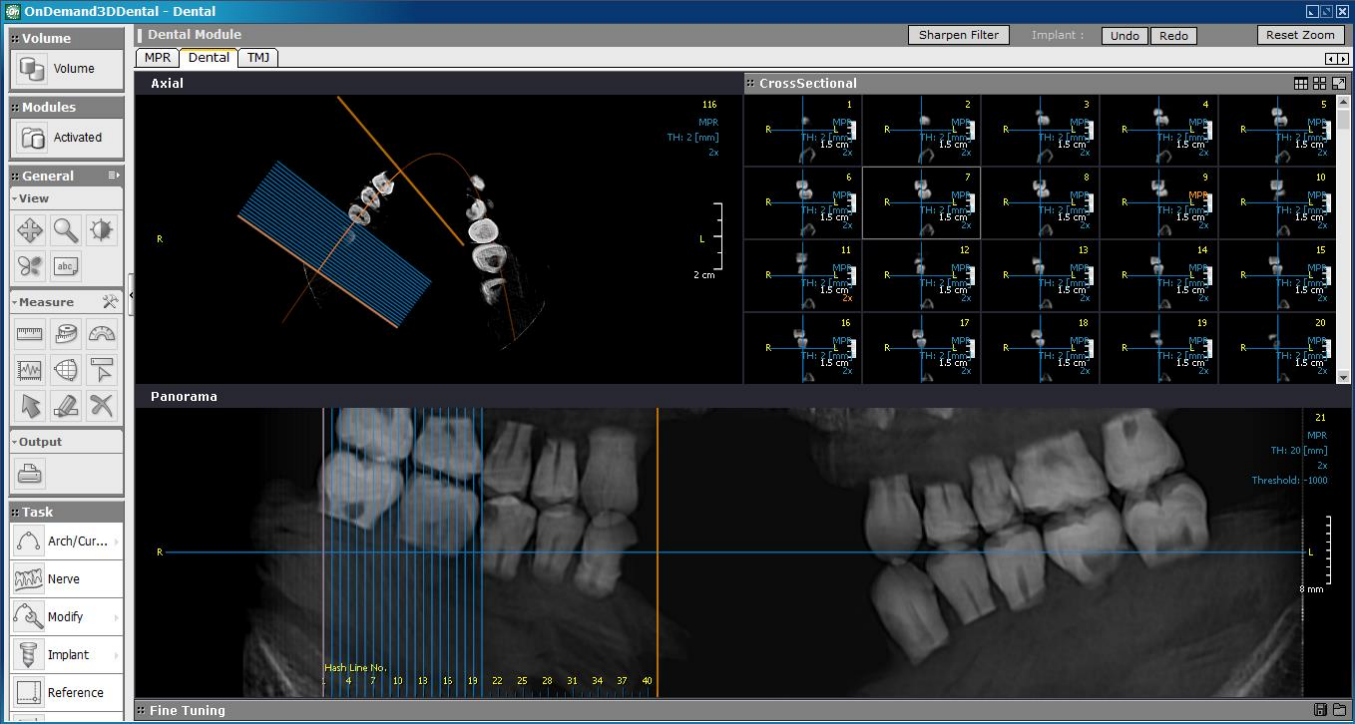
Cone-beam computed tomography (CBCT) image of the skull containing the teeth

The volumetric data were reconstructed using the dedicated software (OnDemand3D Dental 1.0.9.1343, Cybermed Inc., Seoul, South Korea) with the voxel size of 0.075 mm. Figure 2 shows a CBCT image of the skull containing the teeth. The images were then evaluated by two independent oral and maxillofacial radiologists with 6 years of experience, who were blinded to the study sample and had been calibrated during a training session of a pilot study.

The images were viewed on a 22-inch LG Flatron monitor (LG, Seoul, Korea) set at a screen resolution of 1440×6900 pixels and a 32-bit color depth using each system’s specific software in a dimly lit room.

The observers assessed each data set in panoramic (mesiodistal section) and cross-sectional views using three different filtrations (sharpness 0, 1, and 2) and three different slice thicknesses (1, 3, and 5 mm).

The observers scored the absence or presence of occlusal caries using a 5-point scale as follows [21]:

1. caries certainly present
2. caries probably present
3. uncertain-unable to detect
4. caries probably not present
5. caries certainly not present

To assess the intra-observer agreement, the observers evaluated all the images twice with a two-week interval to eliminate memory bias.

The histological validation of the caries status was performed by serially sectioning each tooth mesiodistally parallel to the long axis of the crown using the Accutom-50 (Struers, Ballerup, Denmark). The average thickness of the serial sections was 0.4 mm. Each section was evaluated by an experienced pathologist under a stereomicroscope (Carl Zeiss AG, Oberkochen, Germany) at 15× magnification. The deepest carious lesion in all the sections of a tooth was chosen for scoring that dental surface. Each demineralized white or yellowish-brown discolored area in enamel or dentin was defined as a carious lesion. The assessment of histological sections was performed according to the following scale [22]:

0 = no carious lesion in the occlusal surface
1 = occlusal caries in enamel
2 = occlusal caries extending to the outer half of dentin
3 = occlusal caries extending to the inner half of dentin

**Fig. 3:**
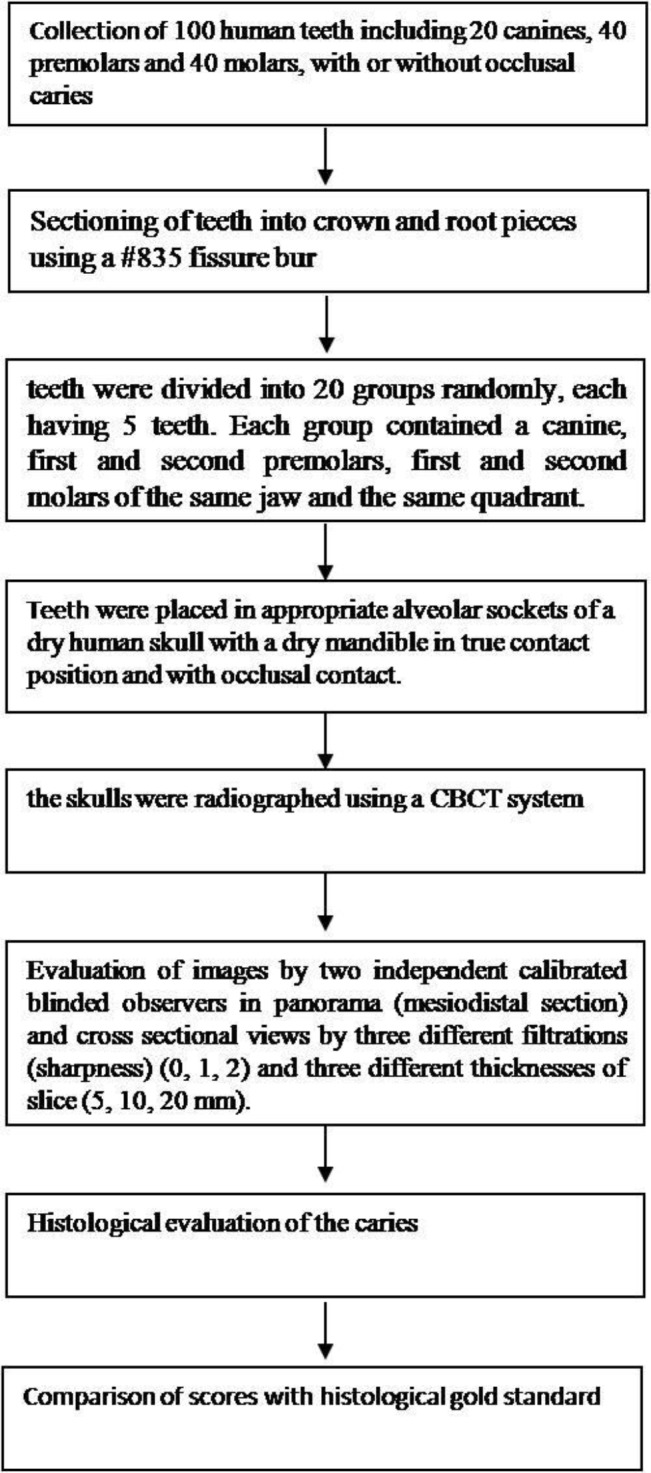
Simple block diagram of the different steps of the methodology for better visualization

All the steps of the methodology are shown in a simple block diagram in Fig 3 for better visualization.

### Statistical analysis:

Weighted kappa coefficients were calculated to assess intra- and inter-observer agreements for each image set according to the following criteria [22]:

<0.10: no agreement
0.11-0.4: poor agreement
0.41–0.60: significant agreement
0.61–0.80: strong agreement
0.81–1.0: excellent agreement

For each observer and each slice thickness and filtration, the parameters expressing various aspects of accuracy, including sensitivity, specificity, positive predictive value (PPV), and negative predictive value (NPV), were computed and validated against the results of the histological examination. Next, the mean of each parameter for both radiologists was computed.

To obtain these accuracy parameters, the radiographic scores from the confidence scale were dichotomized into: Sound (caries definitely absent, caries probably absent, unsure if caries is present or absent) and Lesion (caries probably present, and caries definitely present); this was carried out for scores assigned to both enamel and dentin.

The scores acquired by the radiologists were compared with the histological gold standard using receiver operating characteristic (ROC) analysis to evaluate the diagnostic efficacy. The areas under the ROC curves (Az values) for each slice thickness and filtration were calculated using SPSS software (Version 22.0, IBM Co., Chicago, IL, USA) at α=0.05. Repeated-measures ANOVA and paired t-test were used for comparison between panoramic and cross-sectional views, and significant differences were analyzed binary by the least significant difference (LSD) test.

## RESULTS

The true status of the 100 occlusal surfaces according to histological examinations is presented in Table 1.

**Table 1.** Percentages of carious lesions in each dental region

	** Frequency **	** Percentage **
Without caries	27	27
Caries in enamel	28	28
Caries in the outer half of dentin	24	24
Caries in the inner half of dentin	21	21

According to this table, 27% of the surfaces had no carious lesions, while caries was found in 73% of the surfaces.

The intra-observer kappa coefficients ranged from 0.759 to 0.884 for observer 1 and from 0.716 to 0.867 for observer 2 for different slice thicknesses and filtrations.

Since the inter-observer kappa coefficients ranged from 0.631 to 0.769, we calculated the means of both observers’ readings of each parameter.

The very high values of the intra-observer kappa coefficients suggested strong and excellent intra-observer agreements. Therefore, our calculations were made according to the first reading of each observer.

First, the means of true detection of caries in panoramic and cross-sectional views were assessed. These scores for panoramic and cross-sectional views were 67.77±35.44 and 57.00±26.55, respectively, and the difference was significant (paired t-test, P=0.002). Finally, pairwise comparisons of different filtrations and thicknesses were carried out (Table 2).

**Table 2.** Pairwise comparisons of similar slice thicknesses (mm) and filtrations of cross-sectional and panoramic views (the mean of the two observer's reports)

	** View **	** Mean **	** SD **	** Mean difference 95% CI **		** P-value **

				Lower bound	Upper bound	
** Pair 1 **	Panoramic thickness: 1mm	72.33	38.80	−24.22	−7.78	<0.001
	Cross-sectional thickness: 1mm	88.33	30.84			
** Pair 2 **	Panoramic thickness: 3mm	72.67	36.81	−1.31	15.98	0.096
	Cross-sectional thickness: 3mm	65.33	45.17			
** Pair 3 **	Panoramic thickness: 5mm	58.33	42.74	31.61	50.39	<0.001
	Cross-sectional thickness: 5mm	17.33	37.45			
** Pair 4 **	Panoramic filtration 0	53.33	42.90	−7.95	6.61	0.856
	Cross-sectional filtration 0	54.00	28.73			
** Pair 5 **	Panoramic filtration 1	70.67	38.57	5.85	20.15	<0.001
	Cross-sectional filtration 1	57.67	26.74			
** Pair 6 **	Panoramic filtration 2	79.33	33.43	12.59	27.40	<0.001
	Cross-sectional filtration 2	59.33	26.20			

SD=Standard Deviation, CI=Confidence Interval

**Fig. 4:**
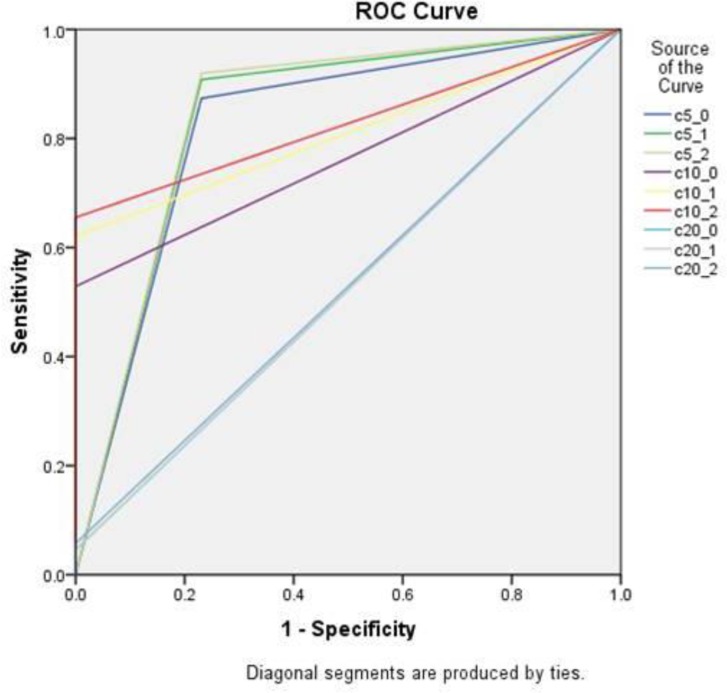
Receiver operating characteristic (ROC) curves of cross-sectional views. (c 5_0= thickness 1 filtration 0, c 5_1=thickness 1 filtration 1, c 5_2= thickness 1 filtration 2, c 10_0= thickness 3 filtration 0, c 10_1=thickness 3 filtration 1, c 10_2= thickness 3 filtration 2, c 20_0= thickness 5 filtration 0, c 20_1=thickness 5 filtration 1, c 20_2= thickness 5 filtration 2)

**Fig. 5:**
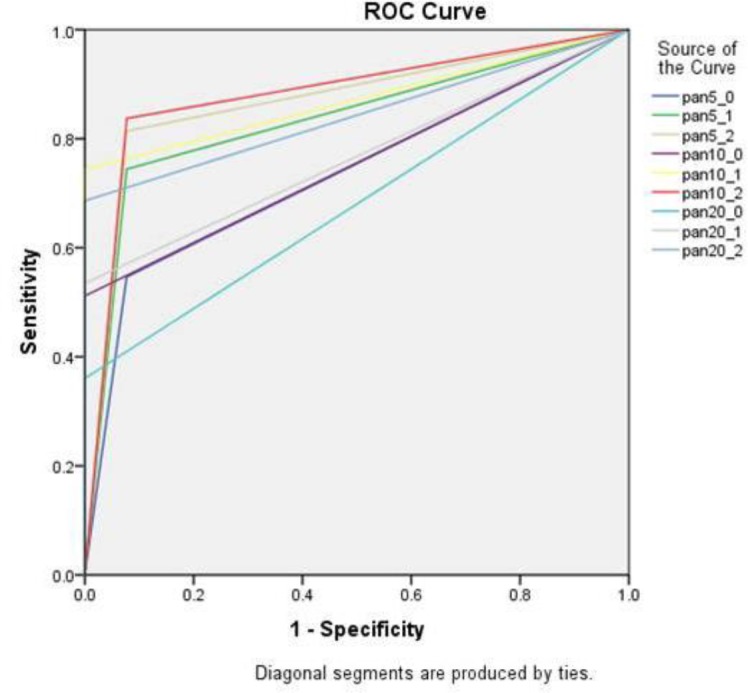
Receiver operating characteristic (ROC) curves of panoramic views. (pan 5_0= thickness 1 filtration 0, pan 5_1=thickness 1 filtration 1, pan 5_2= thickness 1 filtration 2, pan 10_0= thickness 3 filtration 0, pan 10_1=thickness 3 filtration 1, pan 10_2= thickness 3 filtration 2, pan 20_0= thickness 5 filtration 0, pan 20_1=thickness 5 filtration 1, pan 20_2= thickness 5 filtration 2)

Figures 4 and 5 show the ROC curves of cross-sectional and panoramic views.

The mean scores of cross-sectional views were lower than those of panoramic views, except for 1 mm thickness and filtration 0. This difference was significant for all the combinations, except for 3 mm thickness and filtration 0.

Table 3 shows the sensitivity, specificity, PPV, NPV, and Az values of different filtrations and thicknesses. An increase of filtration increased the sensitivity, NPV, and the Az value. All the Az values were greater than the reference point of Az value (0.5), but in 5 mm thickness, this difference was very small. For the evaluation of the effect of slice thickness on the diagnostic accuracy, the mean scores of similar filtrations in panoramic and cross-sectional views were assessed and analyzed using repeated-measures ANOVA (Tables 4 and 5), which showed significant differences in the means of true caries detection in different slice thicknesses of cross-sectional views, but these differences were significant only between 5 mm thickness and other thicknesses in panoramic views; in all the views, increasing the thickness decreased the accuracy of detection. For the evaluation of the effect of filtration on the diagnostic accuracy, the mean scores of similar thicknesses in panoramic and cross-sectional views were assessed and analyzed using repeated-measures ANOVA (Tables 6 and 7), which showed significant differences between different filtrations.

**Table 3.** Sensitivity, specificity, positive predictive value (PPV), negative predictive value (NPV), and Az values of different slice thicknesses (mm) and filtrations (the mean of the two observer's reports)

** Option **	** Sensitivity **	** Specificity **	** PPV **	** NPV **	** Az value **
Panoramic thickness 1, filtration 0	54	92.3	97.9	23.1	0.735
Panoramic thickness 1, filtration 1	73.6	92.3	98.5	34.3	0.834
Panoramic thickness 1, filtration 2	80.5	92.3	98.6	41.4	0.869
Panoramic thickness 3, filtration 0	51.2	100	100	23.6	0.756
Panoramic thickness 3, filtration 1	73.6	100	100	36.1	0.872
Panoramic thickness 3, filtration 2	82.8	92.3	98.6	44.4	0.880
Panoramic thickness 5, filtration 0	35.6	100	100	18.8	0.680
Panoramic thickness 5, filtration 1	52.9	100	100	24.1	0.767
Panoramic thickness 5, filtration 2	67.8	100	100	31.7	0.843
Cross-sectional thickness 1, filtration 0	87.4	76.9	96.2	47.6	0.821
Cross-sectional thickness 1, filtration 1	90.8	76.9	96.3	55.6	0.839
Cross-sectional thickness 1, filtration 2	92	76.9	96.4	58.8	0.844
Cross-sectional thickness 3, filtration 0	52.9	100	100	24.1	0.764
Cross-sectional thickness 3, filtration 1	62.1	100	100	28.3	0.810
Cross-sectional thickness3, filtration 2	65.5	100	100	30.2	0.828
Cross-sectional thickness 5, filtration 0	4.6	100	100	13.5	0.523
Cross-sectional thickness 5, filtration 1	4.6	100	100	13.5	0.523
Cross-sectional thickness 5, filtration 2	5.7	100	100	13.7	0.529

PPV=Positive Predictive Value, NPV=Negative Predictive Value

**Table 4.** Mean scores of similar filtrations in panoramic views

** Panoramic view (Mean±SD) **	** 1 mm thickness **	** 3 mm thickness **	** 5 mm thickness **
1 mm thickness (72.33±38.80)	-	0.900	<0.001
3 mm thickness (72.66±36.81)	0.900	-	<0.001
5 mm thickness (58.33±42.74)	<0.001	<0.001	-

SD=Standard Deviation

**Table 5.** Mean scores of similar filtrations in cross-sectional views

** Cross-sectional view (Mean±SD) **	** 1 mm thickness **	** 3 mm thickness **	** 5 mm thickness **
1 mm thickness (88.33±30.84)	-	<0.001	<0.001
3 mm thickness (65.33±45.17)	<0.001	-	<0.001
5 mm thickness (17.33±37.44)	<0.001	<0.001	-

SD=Standard Deviation

**Table 6.** Mean scores of similar thicknesses in panoramic views

** Panoramic view (Mean±SD) **	** Filtration 0 **	** Filtration 1 **	** Filtration 2 **
Filtration 0 (53.33±42.90)	-	<0.001	<0.001
Filtration 1 (70.67±38.57)	<0.001	-	<0.001
Filtration 2 (79.33±33.43)	<0.001	<0.001	-

SD=Standard Deviation

**Table 7.** Mean scores of similar thicknesses in cross-sectional views

** Cross-sectional view (Mean±SD) **	** Filtration 0 **	** Filtration 1 **	** Filtration 2 **
Filtration 0 (54.00±28.73)	-	0.002	<0.001
Filtration 1 (57.67±26.74)	0.002	-	0.025
Filtration 2 (59.33±26.20)	<0.001	0.025	-

SD=Standard Deviation

LSD test showed that filtration 1 was significantly more accurate than filtration 0, while filtration 2 was the most accurate.

## DISCUSION

In the past decades, the prevalence of dental caries has decreased, but even in populations with low rates of dental caries, the proportion of occlusal caries has increased [23].

American dentists often detect occlusal caries with clinical examination and observation and visual inspection aided by radiographs [24]. Ismail [25] demonstrated that visual and clinical examinations have low sensitivity in detecting occlusal caries. The low sensitivity of clinical examination in detecting caries, especially noncavitated occlusal caries, shows that new methods are necessary for this respect [26]. Several studies have shown that there is no difference between conventional and digital intraoral radiographic techniques in caries detection [10,27]. CBCT produces 3D images with the least distortion and a low exposure dose compared to CT [11,12].

The efficacy of CBCT images in detecting occlusal caries is not clear, and there is no study on the effect of filtration (sharpness) and slice thickness on the caries detection accuracy of CBCT. Qu et al [28] concluded that the type of CBCT system or the FOV does not have any impact on the detection accuracy of approximal caries; therefore, in the present study, only one CBCT system with a fixed FOV and detector was used. In the present study, both inter- and intra-observer agreements were high (strong to excellent), demonstrating that observers were experienced, well-trained, and calibrated before observations.

The results of the present study showed that an increase of filtration increases the sensitivity, while an increase in the thickness results in a lower sensitivity of CBCT imaging in both panoramic and cross-sectional views; however, the specificity was unvarying.

In this study, ROC analysis was used to evaluate the diagnostic accuracy of different slice thicknesses and filtrations. ROC analysis reflects the diagnostic performance more comprehensively than sensitivity and specificity that are determined by only one cut-off point [29]. Moreover, ROC analysis is the most eloquent approach to compare the diagnostic performance of two or more different imaging modalities because it differentiates the inherent capacities of the observers to under- or over-read upon interpreting the images, and it has been used in several studies [30,31].

In the present study, the Az values were between 0.523 and 0.880. To interpret these results, an area of 1 represents a perfect test, whereas anything near 0.5 indicates a poor test result [32]. The application of this standard to our results indicated that CBCT could be considered very accurate, except for different filtrations of 5 mm thickness of cross-sectional views with an Az value of about 0.5.

The outcomes of the present study showed that the panoramic view of CBCT has a higher accuracy in the detection of occlusal caries compared to cross-sectional views; this can be attributed to the fact that showing a complete view of a tooth in the panoramic view allows for an efficient caries diagnosis, while the cross-sectional view shows the tooth partially, which makes it difficult to diagnose carious lesions. Also, in cross-sectional views, occlusal or proximal caries detection is difficult because of the superimposition of proximal and occlusal caries.

In addition, the results of our study revealed that increasing the slice thickness decreases the accuracy of occlusal caries detection. This result was significant with regard to 5 mm thickness of the panoramic view and for all the thicknesses of cross-sectional views. On the other hand, the results indicated that increasing the filtration increases the accuracy of caries detection.

Wenzel et al [33] compared the accuracy of root fracture detection with the use of PSP and CBCT with different filtrations. Their results showed that increasing the filtration in CBCT increases the accuracy of detection of root fracture. Therefore, the results of the present study are consistent with those of the above-mentioned study. Another study showed that increasing the filtration of digital imaging decreases the accuracy of detection of root fracture [18]. This finding is contrary to the results of the present study. The reason behind this difference could be related to the difference in the imaging technique in CBCT and intraoral digital radiography, to a difference in the imaging angle, or to a difference in the exposure dose. Furthermore, the detection of fracture line requires exposure of x-ray in the line of fracture; otherwise, caries detection is simple and does not need accurate x-ray radiation [11].

According to the ALARA principle, radiographic examinations must be fully justified, and evidence-based selection criteria should be considered [19]. Given the overall high Az values, the present study demonstrated the advantage of CBCT in the diagnosis of occlusal caries.

One of the limitations of the current study was that we examined a limited number of slices, while other slices with other thicknesses remained unexamined. Another limitation was that the present study was performed in vitro under ideal imaging conditions without object movements, metallic restorations, any tissue around the teeth, or other variables that can complicate the diagnosis of caries in vivo. Another limitation of this study was the lack of similar studies to compare the results with. It is recommended to perform similar studies with different CBCT systems and larger sample sizes and under in vivo conditions to obtain more accurate results.

## CONCLUSION

Considering the limitations of the present study, it seems that an increase of filtration of CBCT images increases the accuracy of occlusal caries detection; however, an increase in slice thickness results in a lower diagnostic accuracy. Also, the results showed that panoramic views of CBCT are more accurate than cross-sectional views.
